# Delivery of Iron Oxide Nanoparticles into Primordial Germ Cells in Sturgeon

**DOI:** 10.3390/biom9080333

**Published:** 2019-08-01

**Authors:** Abdul Rasheed Baloch, Michaela Fučíková, Marek Rodina, Brian Metscher, Tomáš Tichopád, Mujahid Ali Shah, Roman Franěk, Martin Pšenička

**Affiliations:** 1 University of South Bohemia in Ceske Budejovice, Faculty of Fisheries and Protection of Waters, South Bohemian Research Center of Aquaculture and Biodiversity of Hydrocenoses, Zatisi 728/II, 389 25 Vodnany, Czech Republic; 2 University of Vienna, Department of Theoretical Biology, Althanstraße 14, 1090 Vienna, Austria

**Keywords:** acipenser, caviar, hyperthermia, iron oxide nanoparticles, micro-CT, sterilization

## Abstract

Nanoparticles are finding increasing applications in diagnostics, imaging and therapeutics in medicine. Iron oxide nanoparticles (IONs) have received significant interest of scientific community due to their distinctive properties. For the first time, we have delivered IONs into germ cells in any species. Our results showed that sturgeon primordial germ cells (PGCs) delivered with IONs could be detected until seven days post fertilization (dpf) under fluorescent microscope and at 22 dpf by micro-CT. Delivery of IONs into cells could be helpful for studying germ cell biology and the improvement of germ cell-based bio-technologies as isolation of PGCs using magnetic activated cell sorting or application of hyperthermia for a host sterilization purpose. Intriguingly, in our study, we did not find any toxic effects of IONs on the survival and hatching rates of sturgeon embryos when compared with embryos injected with FITC-dextran only.

## 1. Introduction

Nanoparticles (NPs) are an agglomeration of atoms and molecules ranging from 1 nm to 100 nm, and can be composed of one or more species of the atoms [1]. As a result of their comparable comparable to viruses, cells, genes and proteins, the NPs opened new research avenues in interacting with fundamental biological processes [2]. The NPs are generally classified based on their morphology, composition, agglomeration, dimensionality and uniformity [3]. NPs are made-up of metal, metal oxides, carbon and organic matter, and possess unique magnetic, optical and chemical properties. NPs have been studied thoroughly in different research fields and have generated intense scientific interest in biomedical, optical and electronic fields because of their potential applications [4]. Characterization of NPs have been improved that in-turn has enhanced their applications [5]. Broadly, NPs are divided into two groups, i.e., ultrafine NPs and engineered NPs, which are present in nature and produced in a controlled way, respectively [6].

The engineered magnetic nanoparticles (MNPs) are composed of iron, cobalt or nickel oxides; these particles exhibit special properties like the higher magnetic moment and higher surface to volume ratio; thus enabling them to be potentially manipulated by an external magnetic field [7]. MNPs that are composed of ferromagnetic material, i.e., iron oxide nanoparticles (IONs), made-up of magnetite (Fe_2_O_4_) and maghemite (γ-Fe_2_O_3_) combine the ideal bio-compatibility with superparamagnetic properties, therefore allowing widespread biomedical uses like hyperthermia, targeted drug delivery, biosensors, photoablation therapy, and also in the theranostics applications [8,9]. Moreover, the IONs are also being used in the MRI (magnetic resonance imaging) contrast, and labelling of biological materials [10]. In these applications, iron is not the choice; however, the iron oxides used are more amenable to buffer aqueous environments present in the biological systems. Nevertheless, iron presents advantages over its oxides of being a softer magnet; therefore, it is capable of maintaining its superparamagnetism at larger sizes [11]. The superparamagnetic IONs can be used to improve the treatment of cancer by generating local heat called hyperthermia when exposed to the alternate magnetic field. In hyperthermia as a medical treatment, raising temperature to approximately 43 °C for 30 min to 60 min can trigger apoptosis [12,13]. Tumors as compared to normal cells are more susceptible to hyperthermia due to faster cell division, low pH, increased hypoxia and limited temperature regulation because of poor fluid transfer [14,15]. Additionally, IONs have been shown to be less toxic when compared with other metal oxide NPs [16]. Development of these non-invasive, highly sensitive techniques can be helpful to label any cells (here in our study, germ cells for the first time in any species) will certainly provide knowledge about poorly understood mechanisms [17,18].

Sturgeons, also called as archaic giants are ancient fish species, which have existed for a minimum of 200 million years [19]. As a result of the high value of caviar, the sturgeons became a target of intensive legal and illegal fisheries, therefore resulting in the collapse of several sturgeon species and stocks [20,21]. Natural populations of these living fossils have been declining especially because of the water pollution and interference in their natural habitats. Hybridization, water divergence, reduced food supply and saltwater intrusion are other prominent reasons affecting the populations of sturgeons [22,23]. Moreover, damming of rivers also resulted in the reduction and/or elimination of spawning and egg/larvae habitats of sturgeons [24]. According to the International Union for Conservation of Nature (IUCN) 2010, 85% of sturgeon species are at the verge of extinction. Some sturgeon species have a life span of over 100 years and they attain sexual maturity between 20 to 25 years [21]. However, amongst, the sterlet (*Acipenser ruthenus*) has the fastest reproductive cycle; males mature from three to seven years and females from five to nine years [25]. Thus, this sturgeon species provides opportunities to study the germ cell fate in sturgeons.

The primordial germ cells (PGCs) are the origin of all germ cells in developing embryos that will generate gametes, i.e., spermatozoa and oocyte [26,27]. The formation, migration and proliferation of PGCs are essential for gametogenesis in sexually mature individuals [28,29]. The elucidation of PGCs development in fish species will be helpful to provide fundamental insights regarding gonadal development, sex determination, sexual differentiation [30,31,32] and also a promising technique to manipulate fish reproduction [33,34].

Despite the importance of these amazing ancient fish species, so far not many studies have been conducted regarding the development and PGCs tracking in embryos [27,35]. Previously, our research group have already investigated PGCs development in sturgeon embryos; where visualization of PGCs in sturgeons embryos was done by injecting with a fluorescent tracer dye conjugated to a high-molecular-weight dextran (fluorescein isothiocyanate [FITC]-dextran) [27]. In our present study, however, for first time we have used IONs to label the germ cells in any species; here sturgeons, the IUCN red-listed species, and this study thus can shed light on the interactions of NPs with any cell precisely.

## 2. Materials and Methods

### 2.1. Ethics Statement

All animal experiments were conducted in accordance with the Animal Research Committee of the Faculty of Fisheries and Protection of Waters in Vodňany, University of South Bohemia in České Budějovice, Czech Republic. All experimental fish were maintained according to principles based on the European Union (EU) harmonized animal welfare act of Czech Republic, and principles of laboratory animal care and national laws 246/1992 “Animal Welfare” on the protection of animals were followed. Experiments were approved by the Ministry of Agriculture of the Czech Republic (reference number: MSMT-6406/2019-2).

### 2.2. Fish Source, Preparation of Embryos and Sample Collection

During the spawning season (February to April 2019), females and males of adult sterlet (*Acipenser ruthenus*) of five to nine years of age were transferred from outdoor ponds into the recirculating aquaculture system installed indoor. Fish were kept in tanks of 4000-L at a mean water temperature of 15 °C. To induce spermiation, male sterlet were injected with the single intra-muscular injection of carp pituitary extract at 4 mg/kg body weight (BW) in the 0.9% NaCl. Sperm collection was done 48 h after injection of hormones and kept at ice at 4 °C until fertilization. Light microscopy was used to assess the motility of spermatozoa that was found to be more than 80% and then were used for the fertilization. In order to stimulate ovulation, the carp pituitary extract was used by intra-muscular injection in two doses (first dose at 0.5 mg/kg BW and second at 4.5 mg/kg BW 12 h after the first injection). Ovulated eggs were collected from three different females from 18 to 20 h after the second injection, and these eggs were inseminated with sperm from two males at 15 °C in the dechlorinated water. In order to remove stickiness, eggs were rinsed three times in 1% tannic acid. One hour later after the fertilization, the chorion membrane (outer layer of eggs) was removed by using forceps. Chorion removed eggs were then transferred into 100 mL dechlorinated tap water with 0.01% penicillin and streptomycin in glass petri dishes. Embryos were incubated at 15 °C in an incubator. Temperature regulation was done at 15 ± 1 °C throughout the experimental period and changing of water was done on a daily basis. Embryos were used for injection of IONs mixed with fluorescein isothiocyanate (FITC)- dextran (molecular weight = 500,000) or FITC-dextran only as control in order to label the PGCs. The procedure was repeated two times so that a total of 600 sterlet embryos from six different females were used for the injection of IONs/FITC-dextran. The same number of embryos were used as the control group injected with FITC-dextran only, the remaining embryos were kept as non-injected controls for further incubation to assess hatching and survival rates.

### 2.3. Microinjection of Iron-Oxide Nanoparticles

Injection ready IONs with Rhodamine B [(10 nm) (IRB-10-02)] were bought (Ocean NanoTech, LLC, San Diego, CA, USA). Glass micropipette was drawn from the glass needle (Drummond, Tokyo, Japan) using the needle puller (PC-10; Narishige, Tokyo, Japan). IONs/ 1% FITC-dextran and/or just 1% FITC-dextran were loaded into the glass capillary and thereafter injected into the vegetal pole of sterlet embryos at 1–2 cell stage at 1–4 h post fertilization (hpf) according to Saito et al. [35]. Microinjection of the embryos was performed under the fluorescent stereomicroscope Leica M165 FC (Leica, Wetzlar, Germany) using the automatic micro-injector (Eppendorf, FemtoJet 4×, Hamburg, Germany) with a pressure of ~100 hPa for 1 s. Each embryo was injected with ~50 nL. Survival and hatching rates, and number of IONs and FITC-labelled PGCs at 4 and 5 dpf were examined in all injected groups from different females. Hatched larvae were fed with artemia and at 22 dpf, larvae from each group were anaesthetized by the tricaine solution and the body cavity was opened, gut was dissected and PGCs position were checked in all injected larvae.

### 2.4. Micro-CT Imaging

Paraformaldehyde (PFA)-fixed samples at 22 dpf were post-fixed in the Karnovsky fixative at least overnight and kept in 70% EtOH. Samples were mounted without further contrast staining in 1% low melting temperature agarose in 200 µL pipette tips, and scanned using the MicroXCT system (Zeiss/Xradia, Berlin, Germany) at the Department of Theoretical Biology, University of Vienna, Austria. X-ray projections were taken with the 10× detector objective, tungsten source at 40 kVp (4 W), and pixel size of 2.1–2.5 µm. Reconstructed virtual sections were analysed in Amira 2019.2 (FEI software, Thermo Fisher Scientific, Berlin, Germany). The entire larvae were visualized either by the volume rendering or maximum intensity projection.

### 2.5. Statistical Analysis

The statistical significance of the injection of IONs/FITC-dextran and FITC-dextran on the number of PGCs was analysed by the Wilcoxon rank-sum test. Logistic regression with post hoc Tukey’s test was used for the analysis of survival rates of the embryos. Statistical tests were performed using the R software (Version 3.5.2; R foundation for Statistical Computing, Vienna, Austria) with a significance level of *p*-value < 0.05.

## 3. Results

### 3.1. Fertilization, Hatching and Survival Rates

**Figure 1:**
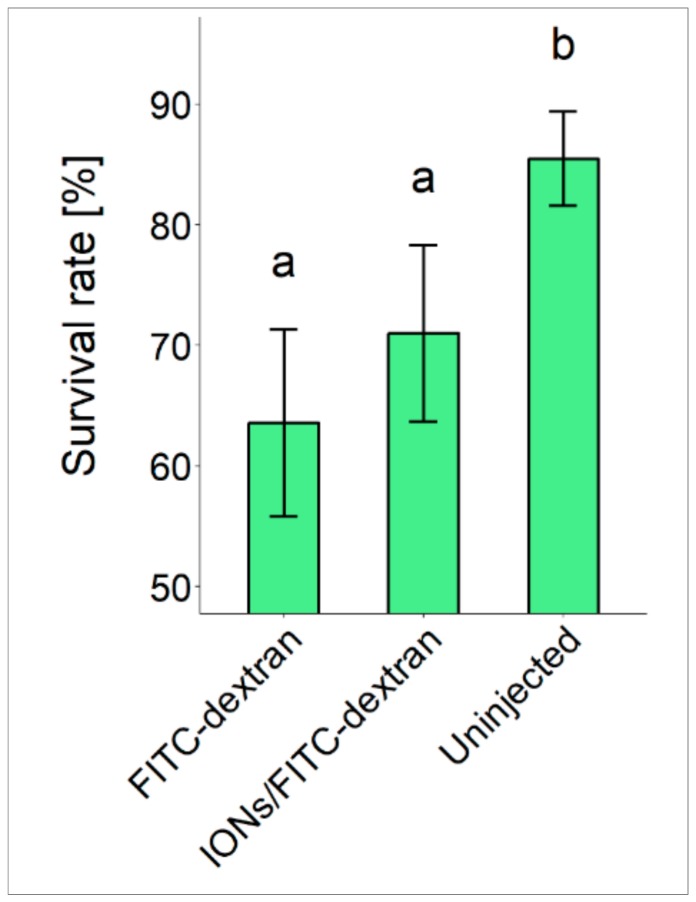
Survival rates of sturgeon embryos injected with FITC-dextran only, IONs/FITC-dextran and uninjected embryos. Different letters (a and b) above the SD bars represent statistical significance.

We used eggs for the injection of IONs and FITC-dextran from six different sterlet females and the fertilization rate of eggs was found to be 95.8 ± 1.8. Hatching and survival rates of sturgeon embryos when injected with IONs are most important indices that help to evaluate the toxicity of IONs. Therefore, we evaluated hatching and survival rates of embryos when injected with FITC-dextran only, IONs/FITC-dextran and un-injected embryos from all six different females (Figure 1). Our data indicates that IONs did not present any toxic effects on the aforementioned parameters when compared with the FITC-dextran injected embryos; however, a significant difference was found when both injected groups were compared with the uninjected group.

### 3.2. Delivery of IONs into PGCs

**Figure 2:**
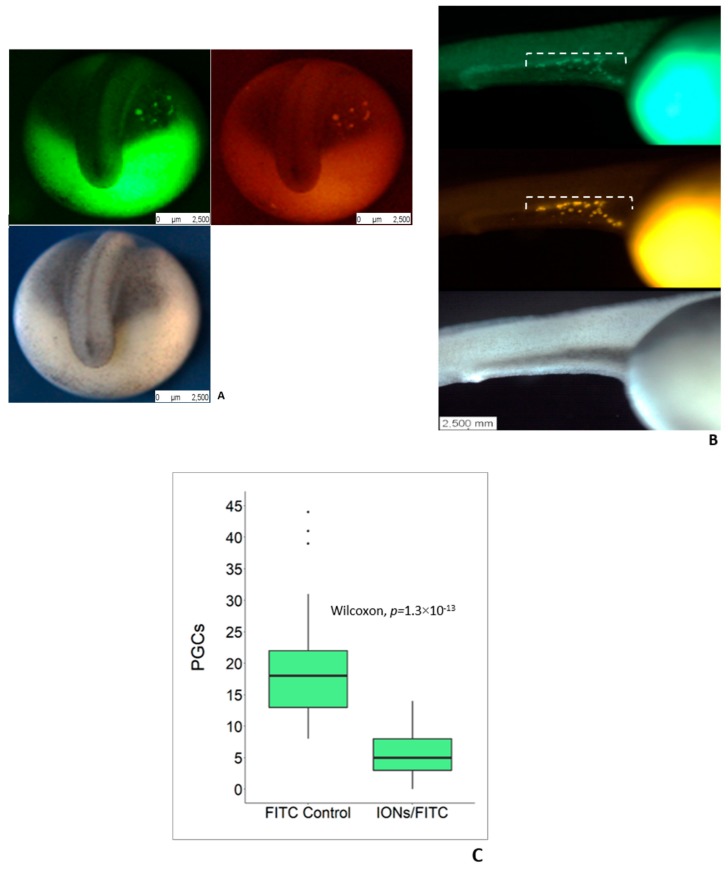
Labelling of primordial germ cells (PGCs) in sturgeons by using IONs and their visualization under fluorescent stereomicroscope. (**A**) IONs/FITC-dextran injected embryos to deliver IONs into PGCs at the tail bud stage. Visualization of PGCs in IONs/FITC-dextran injected embryos at 4 dpf; left: FITC-dextran labelling; right: IONs delivered into PGCs; bottom: Bright field view. (**B**) Visualization of PGCs in IONs/FITC-dextran injected group at 5 dpf. PGCs can be clearly seen under white broken line; top: FITC-dextran labelling; middle: IONs delivered into PGCs; bottom: Bright field view. (**C**) Graph shows a significant difference in the number of PGCs in FITC-dextran injected group and IONs/FITC-dextran injected group; number of PGCs were counted at 4 dpf, (Figure 2c, *p* < 0.05).

We injected IONs mixed with FITC-dextran into the vegetal pole of 1–2 cell stage sturgeon embryos in order to deliver IONs into PGCs according to Saito and Pšenička [27]. PGCs loaded with IONs/FITC-dextran and only FITC-dextran were visualized and they appeared around the margins of tail bud at 4 dpf (Figure 2A). PGCs were also detected at 5 dpf and tracked their migratory pattern that was found to be at the final positon where gonads develop (Figure 2B). The number of PGCs in the embryos injected with FITC-dextran only as the control were found to be in a higher number and significantly differed from those that were injected with IONs/FITC-dextran (Figure 2C).

### 3.3. Micro-CT Imaging

**Figure 3:**
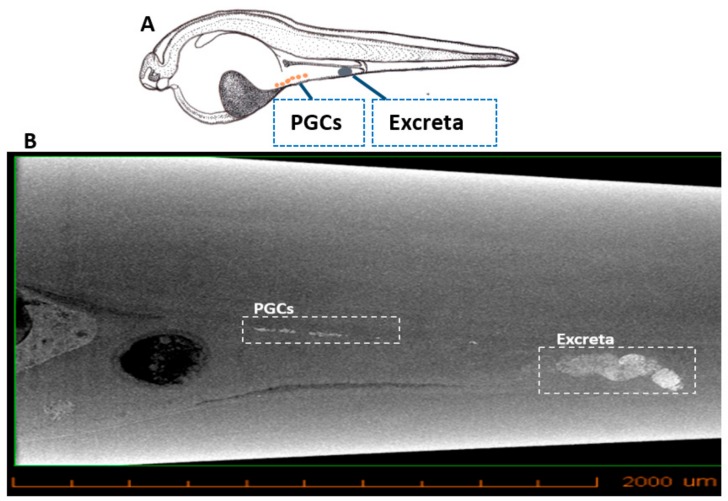
X-ray microtomographic (Micro-CT) section of sturgeon larvae after injection with IONs. (**A**) Illustration showing the position of IONs delivered into PGCs and excreta in sturgeon larvae. (**B**) Micro-CT shows the position of PGCs (in whit dotted box) during their migration towards the position where gonads are formed. Excreta of larvae can also be seen in the white dotted box.

Computed tomography is used for in vivo imaging as it is non-invasive, fast, provides high resolution and cost-effective, and is often employed in research as a micro-CT. IONs injected into sturgeon embryos tend to accumulate in the PGCs due to their enhanced retention capability [36], thus labelling them. The micro-CT imaging showed the PGCs labelled with IONs at 22 dpf (Figure 3; Video S1 in Supplementary Materials).

## 4. Discussion

In our present study, we opted to take the opportunity to use IONs, for the first time to label germ cells in any species. Labelling PGCs by injecting IONs is a non-transgenic approach that provides significant experimental advantages to investigate the biology of germ cells in sturgeons. Transgenic strains such as zebrafish, medaka and trout carrying fluorescent protein in their germ cells have already been produced [37,38,39]. However, on the other hand, sturgeons mature late; their reproduction occurs from five to 25 years, it thus requires 10 to 50 years (at least two generations) to establish transgenic strains. Secondly, the selection and maintenance of transgenic sturgeons will need more keeping space because of big body sizes. In this study, the IONs were injected into the vegetal pole of sterlet embryos from six different females, and no toxic or adverse effects on embryo hatching and survival rates were found after IONs injection. Additionally, we also injected polystyrene NPs into the sturgeon embryos and did not find any toxic effects on survival and hatching rates (data not shown here). It was presumably the no vital somatic tissues in the embryo that were affected with the IONs. Embryos injected with IONs/FITC-dextran and only FITC-dextran were visualized under a fluorescence stereomicroscope to count the number of IONs labelled PGCs from 4 dpf to 7 dpf. We observed a significantly lower number of PGCs labelled with IONs as compared to the control group injected with FITC-dextran only. This could be due to an adverse effect of IONs towards PGCs migration and also FITC-dextran labelling is more effective because molecules of FITC-dextran have a better dispersion capability and thus they have higher labelling efficiency than 10 nm nanoparticles. The injection of IONs did not cause any malformations in embryos. Nevertheless, in zebrafish, when ecological effects of IONs were studied by exposing them against different concentrations, IONs caused developmental toxicity, mortality, malformations and delay in hatching [40].

Similarly to FITC-dextran labelling, according to Saito and Pšenička [27], when the IONs were injected into the vegetal pole, they labelled the PGCs and yolk in the sturgeon embryos; however, yolk is excreted as a faecal material of endogenous nutrition while only PGCs remained labelled. The IONs/FITC-dextran injected embryos were kept until 22 dpf in order to assess the labelling and migratory pattern of PGCs after the first exogenous nutrition intake. However, when the body cavity of euthanized larvae was opened to visualize IONs/FITC-dextran labelled PGCs, only FITC-dextran labelling could be observed under the fluorescent stereomicroscope and the fluorescent signal from IONs (rhodamine B) were lost. Nevertheless, IONs labelled PGCs and excreta could be traced by using micro-CT; and the PGCs were found to be localized at a position where gonadal formation has been described [27], which confirmed the presence of IONs at the transition to exogenous nutrition.

IONs have been studied in connection with hyperthermia to activate cell death [41] by heating up tissues for cancer therapy, and besides, IONs are also released by the cell in vivo as they show no toxic effects [42]. Germ cells labelling in vivo by using IONs can be beneficial to study the interactions of IONs with cells precisely, that will in-turn help how to treat tumors by enhanced generated heat locally with minimal damage to nearby cells or tissues [43]. This application of IONs thus can be applied as an alternate approach to achieve sterility in sturgeon for surrogate production.

Elucidation of the phenomenon of PGCs formation in sturgeons have already been investigated thoroughly by Saito et al. [27] and Saito and Pšenička [35]. They showed that PGCs migration is divided into two phases, i.e., active and slow migration, at different developmental stages. The study was also supported by PGCs localization near mesentery around the hatching stage in Adriatic sturgeons, and PGCs are surrounded by cytoplasmic extensions from somatic cells [44] and thus the fluorescent signal is difficult to be observed. Our present results are consistent with the aforementioned studies and suggest that it will be more convenient to study the interactions of PGCs with somatic cells in sturgeons by using micro-CT after IONs labelling.

The sturgeon PGCs are extremely important cells securing the reproduction of this critically endangered species. However, the number of PGCs in an embryo is very low and handling them is very difficult [35]. An efficient isolation method could enable their research and use in surrogate production technologies. Magnetic-activated cell sorting can be applied when IONs are loaded into PGCs and when the fluorescent signal is difficult to trace under tissues. Efforts and results from our research team have been trying to meet the promise that techniques for ‘‘surrogate production’’ in IUCN red-listed sturgeons are becoming more practicable and convenient [35,45,46,47,48,49].

## 5. Conclusions

We have developed a novel method where IONs were used to label and visualize PGCs in sterlet. PGCs visualization has a great potential for investigation of PGCs development, including their migration and proliferation patterns in developing embryos and hatched larvae. This latest technique can be helpful to study germ cell biology, and consequently the improvement of germ cell based biotechnologies such as PGCs isolation and hyperthermia application for sterilization of host for sturgeon surrogate production.

## Supplementary Materials

The following are available online at https://www.mdpi.com/2218-273X/9/8/333/s1, Video S1: The micro-CT imaging showed the PGCs delivered with IONs at 22 dpf.
